# A first principles study of RbSnCl_3_ perovskite toward NH_3_, SO_2_, and NO gas sensing

**DOI:** 10.1039/d3na00927k

**Published:** 2024-02-05

**Authors:** Mohammad Tanvir Ahmed, Debashis Roy, Abdullah Al Roman, Shariful Islam, Farid Ahmed

**Affiliations:** a Department of Physics, Jashore University of Science and Technology Bangladesh tanvir.phy43@gmail.com; b Department of Physics, Jahangirnagar University Bangladesh

## Abstract

The sensitivity of a RbSnCl_3_ perovskite 2D layer toward NH_3_, SO_2_, and NO toxic gases has been studied *via* DFT analysis. The tri-atomic layer of RbSnCl_3_ possessed a tetragonal symmetry with a band gap of 1.433 eV. The adsorption energies of RbSnCl_3_ for NH_3_, SO_2_ and NO are −0.09, −0.43, and −0.56 eV respectively with a recovery time ranging from 3.4 × 10^−8^ to 3.5 ms. RbSnCl_3_ is highly sensitive toward SO_2_ and NO compared to NH_3_. The adsorption of SO_2_ and NO results in a significant structural deformation and a semiconductor-to-metal transition of RbSnCl_3_ perovskite. A high absorption coefficient (>10^3^ cm^−1^), excessive optical conductivity (>10^14^ s^−1^), and a very low reflectivity (<3%) make RbSnCl_3_ a potential candidate for numerous optoelectronic applications. A significant shift in optical responses is observed through SO_2_ and NO adsorption, which can enable identification of the adsorbed gases. The studied characteristics signify that RbSnCl_3_ can be a potential candidate for SO_2_ and NO detection.

## Abstract 2

The sensitivity of a RbSnCl_3_ perovskite 2D layer toward NH_3_, SO_2_, and NO toxic gases has been studied *via* DFT analysis.

## Graphical abstract

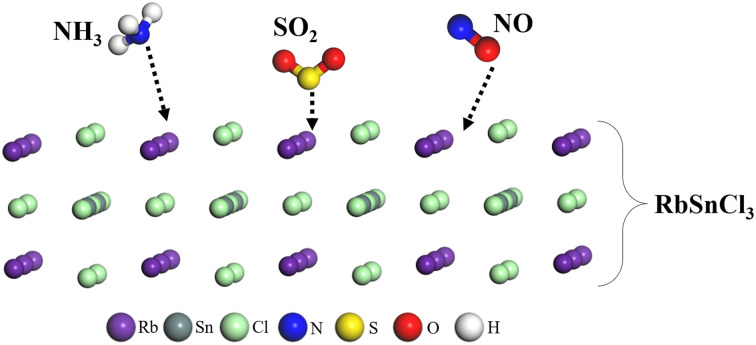

## 1. Introduction

The rapid industrialization and growth of motorized traffic increase the emission of various toxic gases like NH_3_, SO_2_, NO, NO_2_, O_3_, CO, COCl_2_, *etc.* through numerous sources.^1–4^ Among various toxic gases, NH_3_, NO, and SO_2_ are quite soluble in water, and hence they can easily be absorbed into the body through the lungs or skin contact. Once in the body, they can dissolve in the bloodstream and cause harm to organs and tissues. They can also react with other environmental gases to form more harmful gases.^5–7^ NH_3_ is a highly toxic gas with a pungent smell that can be produced by agricultural activity as well as industrial wastes.^1,8^ SO_2_ gas is mainly generated by the burning of various fossil fuels, which is seriously injurious to health and the environment.^2,8^ NO is a highly hazardous gas which can cause even death by asphyxia.^9^ Hence, monitoring these gases is a vital task in order to provide a better living environment, which motivates researchers to create cutting-edge methods of sensing these substances.

Since the historic discovery of graphene, scientists have become more interested in two-dimensional materials.^10,11^ Although graphene is a revolutionary discovery, pure graphene is not suitable for gas detection due to its poor sensitivity.^2^ Previous research revealed that NH_3_ is strongly adsorbed in defected BN NSs, Möbius BCN, graphdiyne NSs, C_2_N_2_ NSs, Pd-doped MoS_2_, CuO/WS_2_ heterostructures, noble metal doped MoSe_2_, *etc.*^1,12–16^ SO_2_ gas showed strong interactions with pristine boron nitride (BN) nanosheets (NSs), boron–carbon–nitride (BCN), BNN doped with Al, Si, Co and Mn, Ti-doped gallium nitride (GN) NSs, Al-doped MoS_2_, and so on.^2,17–21^ On the other hand, germanene NS, silicene NS, C-doped GN NS, aluminene NS, Au-doped MoS_2_, *etc.* 2D layers showed better sensitivity toward NO gas.^22–26^

Perovskites are multifunctional materials with great potential for numerous optoelectronic (OTE) applications.^27^ Various organic/inorganic perovskites have shown remarkable toxic gas-sensing properties both computationally and experimentally.^28–36^ Paper-based sensors of CH_3_NH_3_PbI_3_ (MAPI) perovskite showed a high sensitivity toward NH_3_ gas.^28^ NbWO_6_-based perovskite thin sheets showed strong interaction with H_2_S gas at low temperatures.^29^ ZnSnO_3_ perovskite nanospheres were demonstrated to be potential candidates for *n*-propanol sensors.^30^ Zhuang *et al.* fabricated a SCN-doped MAPI thin film, which showed remarkable sensitivity toward NO_2_ gas.^31^ Balamurugan and Lee synthesized YMnO_3_ nanopowder which demonstrated a fine sensing performance for H_2_S gas.^32^ Liu *et al.* studied the sensitivity of CsPbX_3_ (X = I, Br, and Cl) *via* density functional theory (DFT) calculation, which revealed strong adsorption of CH_2_O gas on CsPbBr_3_.^33^ Aranthady *et al.* reported that a La_0.6_Ca_0.4_FeO_3_ perovskite thin film showed enhanced sensitivity for SO_2_ gas detection.^34^ According to the experimental report of Marikutsa *et al.*, BaSnO_3_ nanocrystals showed high sensitivity for SO_2_ gas.^35^ Formamidinium (FA) lead iodide-based sensors showed strong sensitivity and high selectivity for NH_3_ gas.^36^ RbSnCl_3_ perovskites are reported to be potential candidates for solar cells and thermoelectric and photocatalytic applications.^37,38^ The sensing performance of RbSnCl_3_ for various gases is yet to be studied.

Here we designed a 2D RbSnCl_3_ perovskite with three atomic layers and studied its structural, electronic and optical properties *via* DFT calculations. We also studied the adsorption phenomenon of NH_3_, SO_2_, and NO toxic gases on the RbSnCl_3_ layer. The sensitivity of the RbSnCl_3_ layer toward the selected gases is understood *via* the variation in distinct properties of the perovskite.

## 2 Computational details

In the current study, we have used the CASTEP code to find the global minimum structure and understand the sensing behavior implemented in “Materials Studio”. All of our calculations are performed based on density functional theory with the plane wave basis energy cut-off set to 650 eV. The global minimum structure used a 2 × 2 × 1 *k*-point mesh with the Perdew–Burke–Ernzerhof (PBE) formulation of the generalized gradient approximation (GGA) is used as the exchange–correlation functional,^39^ since GGA-PBE has provided satisfactory results in the study of adsorption and optoelectronic properties.^40–42^ We have set the convergence energy at 1.0 × 10^−5^ eV per atom for the geometry optimization, the ionic displacement at 0.001 Å, and the Gaussian smearing at 0.05 GPa for stress. The Hellmann–Feynman force for every atom has been set at a value of 0.03 eV Å^−1^. We have designed a triatomic layer of RbSnCl_3_ perovskite by building a 2 × 2 × 1 supercell. To avoid the interaction with surroundings we have used a vacuum slab of 35 Å. All complexes are optimized using the same optimization criteria.

The adsorption energy (*E*_ad_) and recovery time (*T*_R_) of the complex structures are calculated from the following equations.

1 *E*_ad_ = *E*_Gas+RbSnCl_3__ − *E*_RbSnCl_3__ − *E*_Gas_,

2
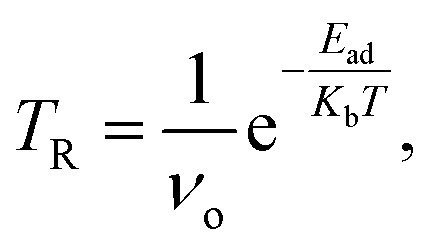
where *E*_Gas+RbSnCl_3__, *E*_RbSnCl_3__, *E*_Gas_, *ν*_o_, *K*_b_, and *T* represent the energy of the gas adsorbed perovskite layer, the energy of the perovskite layer, the energy of isolated gas molecules, incident frequency of UV radiation (*ν*_o_ = 10^12^ Hz), Boltzmann constant, and operating temperature (298 K).

## 3. Results and discussion

### 3.1. Geometry analysis

**Fig. 1:**
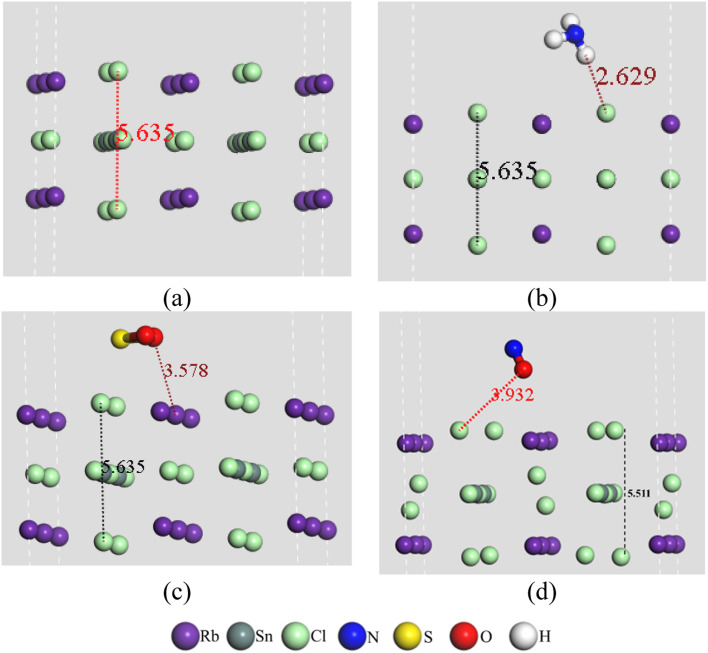
Geometry of (a) RbSnCl_3_, (b) RbSnCl_3_ + NH_3_, (c) RbSnCl_3_ + SO_2_, and (d) RbSnCl_3_ + NO structures.

Fig. 1 shows the optimized geometries of the pristine RbSnCl_3_ layer along with their gas adsorbed complexes. The lattice parameters of the optimized structure are shown in Table 1. The RbSnCl_3_ layer possessed an orthorhombic phase with lattice parameters analogous to those in a previous study.^43^ A slight deformation of the structure is observed due to the adsorption of NH_3_ gas, which suggests a poor interaction of NH_3_ with the adsorbent layer. However, for SO_2_ and NO adsorption, a significant structural deformation is observed, signifying a strong adsorbate–adsorbent interaction. The volume of the RbSnCl_3_ unit cell increased due to the interaction with SO_2_ and NO gas, whereas in the presence of NH_3_, the volume is slightly decreased.

**Table 1:** Lattice parameters of the optimized RbSnCl_3_ before and after gas adsorption

Structures	*a* (Å)	*b* (Å)	*c* (Å)	*α* (degree)	*β* (degree)	*γ* (degree)	*V* (Å^3^)
RbSnCl_3_	5.490	5.490	5.635	90	90	90	169.84
RbSnCl_3_ + NH_3_	5.490	5.489	5.635	90	90	90	169.81
RbSnCl_3_ + SO_2_	5.540	5.490	5.635	85.9	89.9	89.9	171.39
RbSnCl_3_ + NO	5.590	5.540	5.511	90.9	90.6	89.9	170.67

The average bond lengths between atoms are shown in Table 2. The values in the parenthesis represent the bond lengths before adsorption. It is observed that the gas molecules suffer a slight deformation through the adsorption process. The average Sn–Cl bond length of the SnCl_6_ octahedra is about 2.77 Å, which agrees with a previous study.^44^ The average Sn–Cl bond lengths vary slightly due to the interaction of NH_3_ with RbSnCl_3_, whereas a significant structural deformation of the SnCl_6_ octahedra is a result of the interaction with SO_2_ and NO gas molecules.

**Table 2:** The average bond lengths (Å) in the gases and perovskite structure

	N–H	S=O	N=O	Sn–Cl
NH_3_	1.030 (1.029)			
SO_2_		1.459 (1.447)		
NO			1.193 (1.194)	
RbSnCl_3_ + NH_3_				2.767 (2.770)
RbSnCl_3_ + SO_2_				2.791
RbSnCl_3_ + NO				2.812

### 3.2. Adsorption of NH_3_, SO_2_, and NO gases

The adsorption energy, recovery time, and adsorption length (*L*_ad_) are displayed in Table 3. The NH_3_ gas is very weakly adsorbed on the RbSnCl_3_ surface resulting in a very small recovery time of 3.4 × 10^−8^ ms, which is not suitable for practical applications at room temperature. SO_2_ gas is adsorbed significantly by the perovskite layer, whose recovery time makes RbSnCl_3_ suitable for SO_2_ sensing. Among the three selected gases, NO shows the strongest adsorption on the RbSnCl_3_ surface, with a recovery time of 3.5 ms. The strong interaction of NO and SO_2_ with adsorbent can result from the presence of high electronegative elements N, O and S, which can offer strong interaction *via* partial charge transfer between the adsorbent and adsorbate. Among them, NO possesses an unpaired electron in the π* antibonding orbital, which makes it more susceptible to bonding with the adsorbent surface, resulting in the strongest interaction. On the other hand, no unpaired electron is available in NH_3_. Though N is significantly electronegative, the presence of highly electropositive H atoms can generate an opposite force N atom on the adsorbent resulting in weakening of interaction strength.

**Table 3:** Adsorption energy, adsorption length, and recovery time of the complexes

Complexes	Adsorption energy (eV)	Adsorption length (Å)	Recovery time (ms)
RbSnCl_3_ + NH_3_	−0.090	2.629	3.4 × 10^−8^
RbSnCl_3_ + SO_2_	−0.433	3.578	2.06 × 10^−2^
RbSnCl_3_ + NO	−0.564	3.932	3.5

RbSnCl_3_ perovskite showed comparatively poor sensitivity toward NH_3_ gas compared to BC_3_,^45^ graphene,^46^ phosphorene,^47^ silicene, BNNS,^48^ and MoSe_2_ (ref. 49) 2D layers. On the other hand, SO_2_ adsorption energy has a comparatively higher value than that of graphene,^50^ MoS_2_,^51^ MoSe_2_,^52^ and BNNSs.^2^ The NO adsorption on RbSnCl_3_ perovskite is comparatively stronger than on silicene,^23^ BN nanotubes,^53^ MoS_2_,^26^ and MoSe_2_.^54^

### 3.3. Electronic properties

**Fig. 2:**
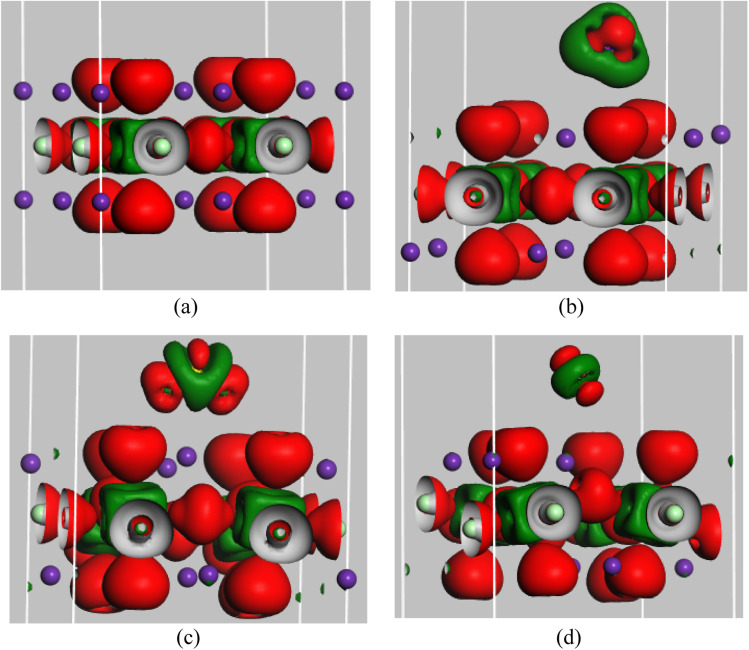
Electron density difference of (a) RbSnCl_3_, (b) RbSnCl_3_ + NH_3_, (c) RbSnCl_3_ + SO_2_, and (d) RbSnCl_3_ + NO structures with an isovalue of 0.02 e Å^−3^.

The interactions between the atoms in pristine RbSnCl_3_ and gas-adsorbed RbSnCl_3_ can be well understood with the help of the electron density difference map and Mulliken (or Hirshfeld) population of the corresponding atoms. Tables 4 and 5 show the Mulliken and Hirshfeld charge distribution of the adsorbent and the complex structures, respectively. In Fig. 2 the red region indicates an electron-enriched region, while the green region shows an electron depletion region. The average Mulliken charge on each Rb, Sn and Cl is +0.87|*e*| +0.67|*e*| and −0.6|*e*| respectively. The A-site element, Rb, shows a partially positive charge due to high electropositivity, and hence Rb atoms act as electron donors. On the other hand, the Cl atoms of the SnCl_6_ octahedra show a partially negative charge due to Cl's higher electronegativity than Sn suggesting the displacement of bonding electrons of Sn–Cl bonds.^55^ The significant amount of the charge transfer indicates a strong covalent bonding between Sn and Cl atoms.^56^

**Table 4:** Average Mulliken charges of the elements in the unit of |*e*|

Structures	Cl	Rb	Sn	N	O	S	H
RbSnCl_3_	−0.600	0.870	0.670				
RbSnCl_3_ + NH_3_	−0.600	0.880	0.670	−1.160 (−1.23)			0.360 (0.41)
RbSnCl_3_ + SO_2_	−0.606	0.853	0.745		−0.800 (−0.80)	1.500 (1.61)	
RbSnCl_3_ + NO	−0.614	0.888	0.680	0.130 (0.14)	−0.120 (−0.14)		

**Table 5:** Average Hirshfeld charges of the elements in the unit of |*e*|

Structures	Cl	Rb	Sn	N	O	S	H
RbSnCl_3_	−0.260	0.380	0.311				
RbSnCl_3_ + NH_3_	−0.258	0.372	0.305	−0.290 (−0.29)			0.080 (0.1)
RbSnCl_3_ + SO_2_	−0.259	0.351	0.338		−0.210 (−0.21)	0.390 (0.41)	
RbSnCl_3_ + NO	−0.263	0.35	0.345	0.031 (0.01)	−0.010 (−0.01)		

After the adsorption of NH_3_, a very slight variation in charge distribution is observed due to the interaction with NH_3_. About −0.08|*e*| charge is transferred from the adsorbent to the NH_3_ molecule suggesting a weak interaction. The average charge on Rb, Sn, and Cl varies significantly due to SO_2_ and NO adsorption. About −0.1|*e*| Mulliken charge is transferred between the adsorbent and SO_2_/NO molecule. The extensive charge transfer between the absorbent and adsorbate through SO_2_ and NO adsorption results in a strong interaction. Hirshfeld charge analysis also verifies the charge transfer. Hence, the variation of Mulliken or Hirshfeld charge is commensurate with the adsorption energy of the three gas molecules under study.

**Fig. 3:**
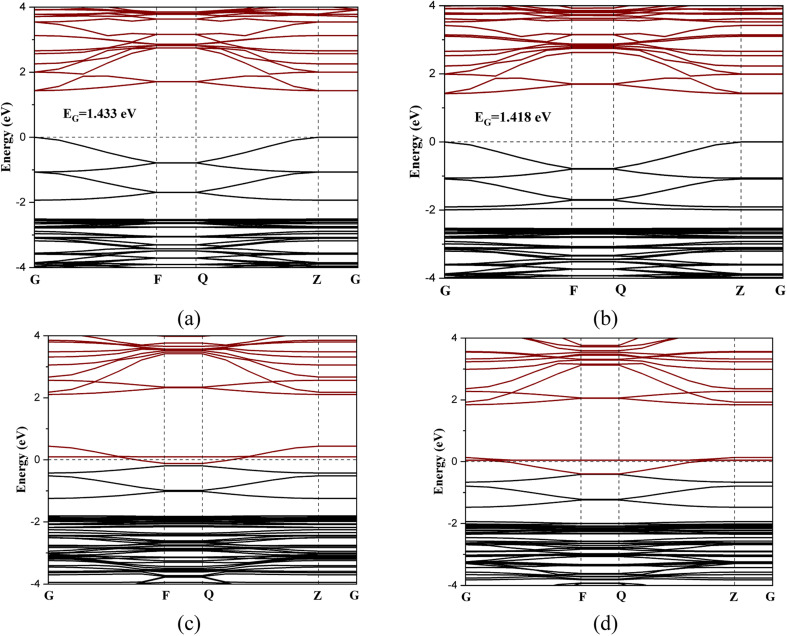
Band structures of (a) RbSnCl_3_, (b) RbSnCl_3_ + NH_3_, (c) RbSnCl_3_ + SO_2_, and (d) RbSnCl_3_ + NO.

Fig. 3 shows the band structures of the perovskite layer and its gas-adsorbed complexes. The RbSnCl_3_ perovskite possesses a direct band gap of about 1.43 eV. Although a similar functional is used in the present study to that previously described, the obtained band gap is significantly higher.^43^ The increase of the band gap can be the result of the quantum confinement effect since in the present study a 2D layer of RbSnCl_3_ perovskite is designed. The band structures are determined along the G → F → Q → Z → G symmetry points of the Brillouin zone. The conduction band minimum (CM) and minimum of the valence band (VM) are located at the ‘*Z*’ *k*-point. The interaction with NH_3_ gas resulted in a slight charge transfer and slight structural deformation, which caused a decrease in the band gap to 1.418 eV. The slight change in the band gap may result in a nominal variation in electrical conductivity, which makes NH_3_ sensing quite harder in practical situations. On the other hand, due to a significant charge transfer and structural deformation, the conduction band of RbSnCl_3_ overlapped with the Fermi level resulting in a zero-band gap while adsorbing SO_2_ and NO gases. Hence, a semiconductor-to-metal transition occurred due to SO_2_ and NO adsorption on the RbSnCl_3_ layer, which can offer a significant change in conductivity resulting in a potential thin layer for SO_2_ and NO sensing. The electrical conductivity (*σ*) of a semiconducting adsorbent is related to the band gap as the following equation,3
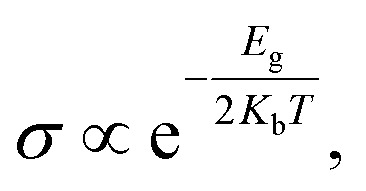


Hence, the conductivity significantly increases after gas adsorption. The absolute measurements of conductivity can be calibrated in order to identify the type of the present toxic gases.

**Fig. 4:**
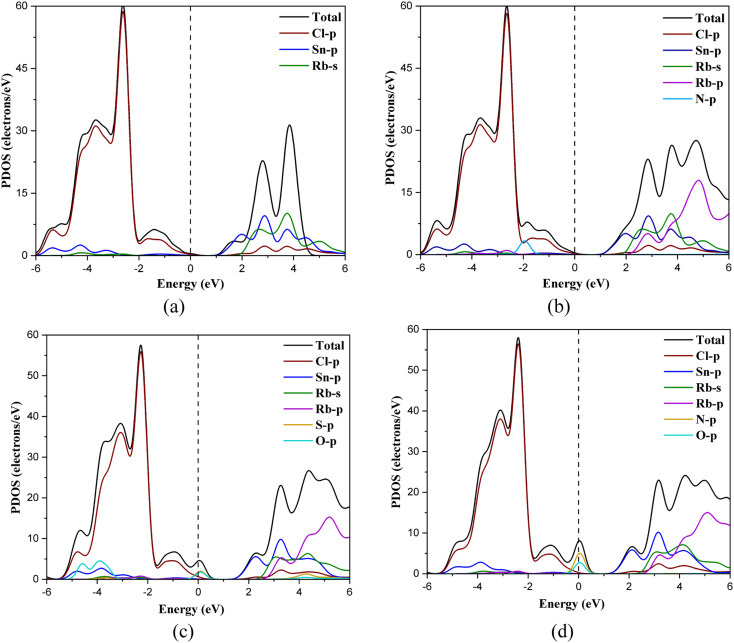
PDOS spectra of (a) RbSnCl_3_, (b) RbSnCl_3_ + NH_3_, (c) RbSnCl_3_ + SO_2_, and (d) RbSnCl_3_ + NO structures.

Fig. 4 shows the partial density of states (PDOS) of the RbSnCl_3_ layer and its gas-adsorbed complexes. In RbSnCl_3_, the electronic configuration of Sn and Cl is 5s^2^5p^2^ and 3s^2^3p^5^ respectively. Hence the contribution of the VM comes from the p-orbital Cl, and the Sn-p orbital contributes to the CM, which is analogous to a previous study.^43^ The A-site element Rb has no significant contribution to the VM or CM, and hence it does not directly affect the crystal band edge, which agrees with previous studies.^57,58^ No significant variation in electronic contribution to the VM and CM is observed due to NH_3_ adsorption, which is consistent with the nominal variation in the band gap. After SO_2_ adsorption, band overlapping was observed due to the contribution of p orbitals of S and O atoms near the Fermi level, which as a result reduced the band gap. A similar phenomenon is observed with NO adsorption. The p-orbitals of both N and O contribute significantly to the Fermi level resulting in a zero-band gap.

### 3.4. Optical properties

**Fig. 5:**
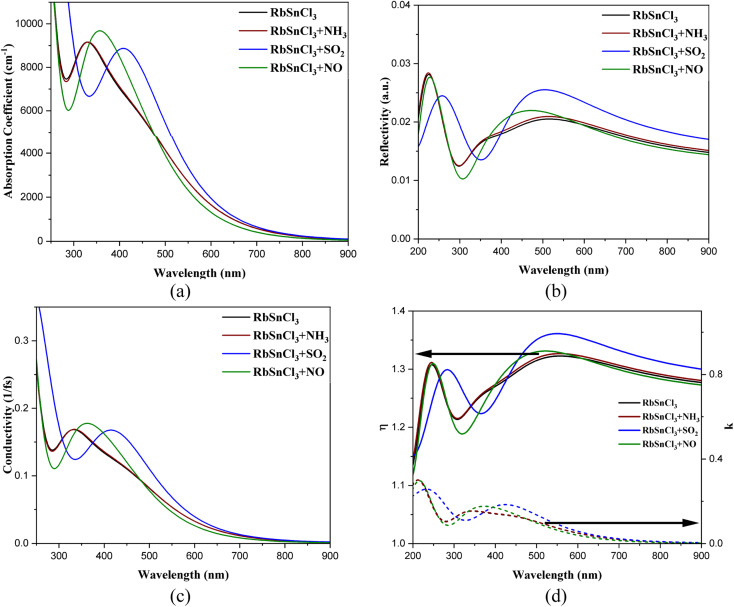
(a) Absorption coefficient, (b) reflectivity, (c) conductivity, and (d) refractive indices of the optimized geometries.

The optical responses of the RbSnCl_3_ perovskite layer in the visible ranges satisfy previous findings.^43^ The RbSnCl_3_ perovskite shows an absorption coefficient (AC) over 10^3^ cm^−1^ order (Fig. 5) in the visible region, which makes it a potential material for various optoelectronic applications. The high AC along with the band gap of 1.43 eV can provide better visible light absorption in solar cells. No significant variation in the AC is observed after NH_3_ adsorption. However, a significant red shift of the absorption peak is observed due to NO and SO_2_ adsorption, which suggests a strong optical response in gas sensing. The shift in the absorption peak can be used in determining the type of adsorbed gas.

An extensive redshift of the reflectivity peak is observed through NO adsorption, which suggests that there would be a change in the color of the RbSnCl_3_ material in the presence of NO gas. This idea can also be applied in sensing and detecting the nature of the toxic gas present in the environment. All the structures showed very poor reflectivity (<3%) in the visible region, suggesting a small portion of energy loss *via* reflection.

RbSnCl_3_ showed high optical conductivity (OC) in the lower wavelength region of the visible spectrum. The OC peak is observed in the near UV region of value over 10^14^ s. The high OC is preferable for various optoelectronic applications, *i.e.*, photodiodes, detectors, *etc.* No observable variation in the OC occurred in the presence of NH_3_ gas; however, a redshift of the OC peak in the presence of NO and SO_2_ suggests a significant optical response in the visible wavelength. Conductivity gradually decreases with increasing wavelength.

The RbSnCl_3_ perovskite shows a low refractive index (*η* = 1.24–1.33) for visible wavelength. A low *η* is always preferable for optoelectronic research and the materials are suitable as antireflection coatings.^59^ A low *η* signifies a low energy loss *via* reflection. NH_3_ adsorption shows no significant variation in the refractive index; however, a drastic change is observed *via* SO_2_ and NO adsorption. The *η* increased up to 1.36 due to NO adsorption. The imaginary part of the refractive index (*k*) is also known as the extinction coefficient. A non-zero value of *k* signifies optical absorption, which increases with increasing the value of *k*. Hence, the *k*-spectra are analogous to the absorption spectra, where *k* is the maximum in the lower wavelength region of the visible spectrum. The value of *k* for RbSnCl_3_ perovskite at 400 nm is 0.14, which further increased to 0.170 and 0.176 after NO and SO_2_ adsorption, respectively. The peak of *k* red shifted due to NO and SO_2_ adsorption with an increase in its value suggesting an increase in absorption in the presence of NO and SO_2_ gases.

The significant variation in optical responses, *i.e.*, shifting of absorption, reflection, and conductivity maxima, can be calibrated in order to identify the nature of adsorbed gases. Since both RbSnCl_3_ + SO_2_ and RbSnCl_3_ + NO are zero band gap complexes, the variation in optical characteristics (color) can be a way to distinguish between SO_2_ and NO adsorption.

## 4. Conclusion

A triatomic layer of RbSnCl_3_ perovskite is designed and optimized to ground state geometry successfully through DFT calculations. The toxic gases NH_3_, SO_2_, and NO are adsorbed on the RbSnCl_3_ layer with negative adsorption energies with a strong interaction between RbSnCl_3_–SO_2_ and RbSnCl_3_–NO. The RbSnCl_3_–SO_2_ and RbSnCl_3_–NO interactions result in comparatively more structural deformation of RbSnCl_3_ crystals than RbSnCl_3_–NH_3_ interaction. The charge transfer between adsorbent and adsorbate also verifies the strong interactions. The recovery times in the ms range also verify the applicability of the RbSnCl_3_ gas sensor in a practical environment. The band gap of the pure RbSnCl_3_ layer is about 1.43 eV which decreased to 1.412 in the presence of NH_3_; however, a complete semiconductor-to-metal transition is observed due to SO_2_ and NO adsorption. The RbSnCl_3_ layer showed a high AC, OC and very low reflectivity endowing it with potential for numerous optoelectronic research studies. A significant red shift in the AC and OC is observed due to SO_2_ and NO adsorption, which can be used to detect the type of adsorbed gas. The strong adsorption energies and variation in electronic and optical properties suggest that RbSnCl_3_ perovskite is a potential gas sensor for detecting as well as identifying NO and SO_2_ toxic gases.

## 5 Future work

The synthesis and gas sensitivity of RbSnCl_3_ perovskite can be determined experimentally. The gas sensitivity of various perovskite structures can be studied both theoretically and experimentally. Various environmental factors, *i.e.*, temperature, pressure, and presence of harmless environmental gases may affect the sensitivity of the gas sensor, which can be investigated in future studies.

## Conflicts of interest

There are no conflicts to declare.

## Supplementary Material
